# HIV drug resistance against strand transfer integrase inhibitors

**DOI:** 10.1186/s12977-017-0360-7

**Published:** 2017-06-05

**Authors:** Kaitlin Anstett, Bluma Brenner, Thibault Mesplede, Mark A. Wainberg

**Affiliations:** 1 Department of Microbiology and Immunology, Faculty of Medicine, McGill University, Montreal, QC Canada; 2 McGill AIDS Centre, Lady Davis Institute for Medical Research, Jewish General Hospital, 3755 Cote-Ste-Catherine Road, Montreal, QC Canada

**Keywords:** HIV, Resistance, Selections, Clinic, INSTIs, Raltegravir, Elvitegravir, Dolutegravir, Cabotegravir, Bictegravir

## Abstract

Integrase strand transfer inhibitors (INSTIs) are the newest class of antiretroviral drugs to be approved for treatment and act by inhibiting the essential HIV protein integrase from inserting the viral DNA genome into the host cell’s chromatin. Three drugs of this class are currently approved for use in HIV-positive individuals: raltegravir (RAL), elvitegravir (EVG), and dolutegravir (DTG), while cabotegravir (CAB) and bictegravir (BIC) are currently in clinical trials. RAL and EVG have been successful in clinical settings but have relatively low genetic barriers to resistance. Furthermore, they share a high degree of cross-resistance, which necessitated the development of so-called second-generation drugs of this class (DTG, CAB, and BIC) that could retain activity against these resistant variants. In vitro selection experiments have been instrumental to the clinical development of INSTIs, however they cannot completely recapitulate the situation in an HIV-positive individual. This review summarizes and compares all the currently available information as it pertains to both in vitro and in vivo selections with all five INSTIs, and the measured fold-changes in resistance of resistant variants in in vitro assays. While the selection of resistance substitutions in response to RAL and EVG bears high similarity in patients as compared to laboratory studies, there is less concurrence regarding the “second-generation” drugs of this class. This highlights the unpredictability of HIV resistance to these inhibitors, which is of concern as CAB and BIC proceed in their clinical development.

## Background

Since the beginning of the pandemic, HIV/AIDS has claimed the lives of over 35 million people, and approximately 35 million individuals are currently infected [1]. Highly active antiretroviral therapy (HAART) has transformed a positive HIV diagnosis from a former death sentence into a chronic, manageable disease. However, no cure yet exists for HIV and patients must remain on therapy for the entirety of their lives which makes the development of drug resistance in the virus a real concern. In fact, drug resistance has been documented for every currently available drug class in patients [2]. This makes the continued study of the mechanisms of HIV drug resistance and novel therapeutics a top priority for HIV scientists worldwide.

The reverse transcriptase (RT) enzyme of HIV is highly error-prone, introducing mutations into the genome at a rate of 1.4 × 10^−5^ mutation per base pair, per replication cycle [3]. This high mutation rate allows for the generation of multiple different viruses within an infected individual, sometimes referred to as “quasi-species.” If one of these quasi-species has a mutation that provides a selective advantage for replication in the presence of antiretrovirals (ARVs), it will out-compete other viral forms to become the dominant species [4].

The integrase (IN) enzyme catalyses the insertion of the viral DNA (vDNA) into the host’s genome through two catalytic actions: 3′ processing and strand transfer. In the cytoplasm, IN self-associates into tetramers on the newly reverse transcribed vDNA, where it catalyzes the removal of the last two nucleotides from the 3′ ends of both strands [5]. In addition, IN can spontaneously form larger multimers that are stabilized by the addition of allosteric integrase inhibitors, and reciprocally destabilized in the presence of DNA [6–10]. After nuclear translocation, IN associates with lens epithelium-derived growth factor (LEDGF)/p75 and is directed to sites of open chromatin, where it will initiate strand transfer, i.e. the nucleophilic attack of the 3′ hydroxyl groups on the viral DNA on the nucleotide backbone of the host DNA. The integration process is completed by host gap-repair machinery, resulting in a 5 base-pair repeat that flanks each end of the viral DNA [11].

The integrase strand transfer inhibitor (INSTI) class of antiretroviral drugs is the latest to be approved for treatment of HIV-positive individuals. As their name suggest, INSTIs inhibit the second step catalyzed by IN, i.e. strand transfer, through competitive binding to the enzyme’s active site. INSTIs not only displace the 3′ end of the vDNA from the active site, but also chelate the divalent cation (Mg^2+^ or Mn^2+^) that is required for IN enzymatic activity [12]. There are currently three INSTIs approved for the treatment of HIV infection: raltegravir (RAL), elvitegravir (EVG), and dolutegravir (DTG) [13]. Cabotegravir (CAB) and bictegravir (BIC) are newer INSTIs currently in clinical trials [14, 15].

Although highly efficacious in the management of HIV, both RAL and EVG are susceptible to virological failure through the development of resistance mutations. What is more, most of the changes that cause resistance to RAL also cause resistance to EVG, and vice versa [16]. This is, however, not the case with DTG. Not only does DTG appear to have a higher genetic barrier to resistance than either of the other two drugs, it has not yet been shown to definitively select for any resistance-associated changes in treatment-naïve patients [17]. Although two reports of potential emergence of resistance in individuals treated with DTG in first line therapy recently appeared, baseline IN was not sequenced in one of these cases, nor did the supposed-emergent mutation lead to persistent virological failure while DTG was still being used together with an optimized background regimen containing rilpivirine (RPV), an NNRTI with a modest genetic barrier to resistance [18]. Specifically, initial TDF/FTC/DTG treatment was supplemented with ritonavir-boosted darunavir following failure; the latter drug was subsequently substituted with RPV for reason of diffuse erythoderma. The second case reported transient emergence of a T97A substitution that did not confer any resistance on its own against DTG in vitro and was not observed at subsequent time points [19]. Although it cannot be excluded that unambiguously documented cases of emergent resistance mutations against first-line DTG will eventually be reported, it is expected that this will be rare. This is supported by the fact that despite dolutegravir being used by tens of thousands treatment-naïve individuals in Europe and the USA, the abovementioned two cases are the only known reports of potential primary de novo resistance against this drug. There have also been rare cases of treatment failure with resistance mutations in treatment-experienced but INSTI-naïve patients, and, in this setting, DTG has most often selected for the novel resistance substitution R263K [20]. Other substitutions at residues E92, Q148 and N155, have been reported when DTG monotherapy was used in treatment-experienced patients.

Primary resistance substitutions arise first in response to INSTI drug pressure and cause a decrease in susceptibility at the expense of viral fitness, most often through alterations to the enzyme’s active site where the inhibitors bind [16, 21]. Secondary resistance substitutions arise after continued drug pressure and usually act to alleviate the negative effects of primary mutations, and may also increase levels of INSTI resistance [22, 23]. Some of these secondary changes are specific to a certain primary resistance pathway, but many may be selected after several different primary mutations.

Pre-clinical and in vitro studies have been instrumental in the evaluation of novel therapeutic agents for the treatment of HIV infection, however they do not always accurately predict clinical outcomes for patients. Laboratory viral strains and cell lines, although excellent scientific tools, can never recapitulate in vivo human infections with 100% accuracy. In this review, we compare both the in vitro selection and antiviral activity reported for drugs of the INSTI class with the analogous data available from treated patients to assess the predictive power of in vitro studies for INSTI clinical outcomes.

## Raltegravir

In 2004 a group of researchers at Merck & Co. reported on the efficacy of the diketo acid (DKA)-based lead compound L-870812 against simian immunodeficiency virus (SIV) in infected rhesus macaques [24]. This led to the approval of the first INSTI, raltegravir, in 2007 for treatment-experienced AIDS patients with multidrug resistance, and two years later for treatment-naïve individuals as well [25, 26]. In the 10 years since its first approval, RAL has been shown to be well tolerated in the vast majority of patients, although it is does require twice daily dosing. It displays a modest genetic barrier to resistance, with the most common mutational pathways consisting of changes at positions Y143, Q148, and N155 [27].

The substitutions in IN that have been selected both in cell culture and in treated patients with RAL are summarized in Tables 1, 2, 3 and 4, and the measured fold-changes in resistance to INSTIs for the different combinations of substitutions in each of the major pathways are displayed in Tables 5, 6, 7 and 8. There were sporadic reports of changes at positions T66 and E92 with RAL, mostly in vitro (Table 1). However, the main resistance pathways that have been reported as selected both in vitro and in vivo with RAL are Y143, Q148, and N155 (Tables 2 and 3). As shown in Table 5, these pathways only provide low to moderate changes in RAL susceptibility, which helps to explain their limited selection. Broadly, there is a high concurrence between the resistance pathways selected both in tissue culture under RAL pressure and in patients undergoing therapy with RAL.

**Table 1:** In vitro and in vivo selection of de novo primarily elvitegravir-associated resistance substitutions in HIV IN in tissue culture and in INSTI-naïve individuals

Substitutions	RAL		EVG		DTG	
Selection	In vitro	In vivo	In vitro	In vivo	In vitro	In vivo
*T66*						
T66A	X	X	X	X		
T66I			X	X		
T66I/V72A/A128T			X			
T66I/E92Q			X			
T66I/E92Q/T124A			X			
T66I/Q95K/E138K/Q146P/S147G			X			
T66I/T97A/G163R	X					
T66I/T124A			X			
T66I/T124A/Q146L			X			
T66I/I203M		X				
T66I/F121Y/S153Y/D232N			X			
T66I/D232N			X			
T66I/R263K			X			
T66K	X		X	X		
T66K/L74M	X					
T124A/T66K	X		X			
*E92*						
E92G			X			
E92Q	X	X	X	X	X	
L74M/E92Q		X				
H51Y/E92Q/S147G			X			
H51Y/E92Q/S147G/E157Q			X			
E92Q/M154I	X					
E92V			X			
E92V/T124A			X			

‘X’ marks a report of the selection of a substitution or combination of substitutions. Numbers refer to amino acid position in HIV integrase, one letter amino acid code used

References: [14, 18–20, 28–53]

**Table 2:** In vitro and in vivo selection of de novo exclusively raltegravir-associated resistance substitutions in HIV IN in tissue culture and in INSTI-naïve individuals

Substitutions	RAL	
Selection	In vitro	In vivo
*Y143*		
Y143C	X	X
L74M/T97A/Y143R/G163R		X
T97A/Y143C/G163R		X
Q95K/Y143C		X
L74M/T97A/E138D/Y143R/G163N		X
T97A/Y143C		X
Y143C/S230R		X
L74M/T97A/Y143G		X
Y143H	X	X
Y143K		X
T97A/Y143S		X
T97A/E138A/Y143K		X
Y143S/V201I		X
Y143R	X	X
V72I/Y143R/T206S		X
L74M/T97A/Y143R		X
L74M/T97A/E138A/Y143R		X
T97A/Y143R		X
Y143R/D232N		X
G140S/Y143R		X

‘X’ marks a report of the selection of a substitution or combination of substitutions. Numbers refer to amino acid position in HIV integrase, one letter amino acid code used

References: [14, 18–20, 28–53]

**Table 3:** In vitro and in vivo selection of de novo pan-INSTI resistance substitutions in HIV IN in tissue culture and in INSTI-naïve individuals

Substitutions	RAL		EVG		DTG		CTG	
Selection	In vitro	In vivo	In vitro	In vivo	In vitro	In vivo	In vitro	In vivo
*Q148*								
Q148H	X	X	X	X				
G140S/Q148H	X	X	X	X				
G140C/Q148H	X	X	X	X				
G140A/Q148H	X	X	X	X				
E138K/Q148H	X	X	X	X				
E138A/Q148H	X	X	X	X				
T112S/G140S/Q148H/G163R		X						
E138A/G140S/Q148H		X						
E138A/G140S/Y143H/Q148H		X						
E138K/G140S/Q148H		X						
G140S/Y143H/Q148H		X						
G140S/Q148H/G163R		X						
G140S/Q148H/S230N		X						
Q148K	X	X	X	X				
N17S/Q148K	X							
N17S/Q148K/G163R	X							
G140S/Q148K	X	X	X	X				
G140C/Q148K	X	X	X	X				
G140A/Q148K	X	X	X	X				
E138K/Q148K	X	X	X	X				
E138A/Q148K	X	X	X	X				
Q148K/G163R	X							
E138A/G140A/Q148K		X						
G140C/Q148K/G163R	X							
E138K/Q148K/G163R	X							
E92Q/E138K/Q148K/M154I	X							
G140S/Q148N				X				
Q148R	X	X	X	X				X
T124A/Q148R	X		X					
H114Y/A128T/Q148R			X					
G140S/Q148R	X	X	X	X				
G140C/Q148R	X	X	X	X				
G140A/Q148R	X	X	X	X				
E138K/Q148R	X	X	X	X				
E138A/Q148R	X	X	X	X				
G140A/Q148R/G163R		X						
Q148R/N155H		X						
E138K/Q148R/G163R		X						
G140S/Q148R/G163R		X						
E138K/Q148R/N155H/G163R		X						
Q148R/N155H/G163R/S230N		X						
L74M/G140A/Q148R		X						
L74M/Q95T/G140A/Q148R		X						
Q148R/D232N	X							
*N155*								
N155H	X	X	X	X		X		
I60L/T97A/N155H						X		
V72I/N155H		X						
L74M/N155H		X						
L74M/Y143R/N155H		X						
L74M/T97A/V151I/N155H		X						
L74I/V151L/N155H		X						
E92A/N155H		X						
E92Q/N155H		X	X					
E92Q/V151I/N155H		X						
E92Q/N155H/G163R		X						
E92Q/N155H/R263K			X					
Q95K/N155H		X						
T97A/N155H		X						
T97A/Y143C/N155H	X							
T97A/V151L/N155H		X						
T97A/V151I/N155H		X						
T97A/V125A/V151I/N155H		X						
T97A/E138D/V151I/N155H		X						
T124A/V151I/N155H	X							
E138D/N155H		X						
E138K/N155H/G163R		X						
Y143C/N155H		X						
V151I/N155H	X							
V151I/N155H/V125A		X						
V151L/N155H		X						
N155H/G163R		X						
N155H/I204T	X							
N155H/R263K			X					
N155S	X							
N155S/D232N	X							

‘X’ marks a report of the selection of a substitution or combination of substitutions. Numbers refer to amino acid position in HIV integrase, one letter amino acid code used

References: [14, 18–20, 28–53]

**Table 4:** In vitro and in vivo selection of de novo non-canonical INSTI resistance substitutions in HIV IN in tissue culture and in INSTI-naïve individuals

Substitutions	RAL		EVG		DTG		CTG		BIC	
Selection	In vitro	In vivo	In vitro	In vivo	In vitro	In vivo	In vitro	In vivo	In vitro	In vivo
*R263K*										
R263K			X		X	X			X	
M50I/R263K					X				X	
A49G/S230R/R263K						X				
M50I/S119R/R263K					X					
H51Y/R263K					X					
S119R/R263K					X					
E138K/R263K					X					
E138AKT/S147G/R263K						X				
V151I/R263K					X					
S153Y/R263K					X					
V260I/R263K						X				
*Other*										
H51Y/G118R					X					
V54I	X									
G59E	X									
L74M		X		X		X				
Q95K	X		X							
T97A		X		X		X				
L101Y/T124A/S153Y					X					
I203M		X								
H114Y			X							
G118R		X			X					
F121Y	X	X	X							
F121Y/G163R	X									
F121Y/D232N	X									
T124A	X		X		X		X			
T124A/P145S			X							
T124A/S153F					X					
T124A/S153Y							X			
T124A/Q146L			X							
T125K		X								
A128T	X		X							
P145S			X							
Q146L							X			
Q146P			X							
S147G			X	X						
V151I	X	X	X			X				
S153Y							X			
M154I		X								
E157Q		X								
I162M							X			
G163E						X				
G163R		X								
Q177R			X							
G193E					X					
S230R	X	X	X							

‘X’ marks a report of the selection of a substitution or combination of substitutions. Numbers refer to amino acid position in HIV integrase, one letter amino acid code used

References: [14, 18–20, 28–53]

**Table 5:** Levels of in vitro resistance for HIV-1 INSTI mutated viruses with elvitegravir resistance pathways

Genotype	RAL	EVG	DTG	CTG	BIC
*T66*					
T66A	−	+	−	−	NA
T66A/S153F	−	++	NA	NA	NA
T66I	−	+	−	−	NA
T66I/L74M	+	++	−	−	NA
T66I/E92Q	++	+++	−	−	NA
T66I/F121Y	+	++	NA	NA	NA
T66I/S153Y	−	+++	NA	NA	NA
T66I/E157Q	−	++	−	NA	−
T66I/Q146P	NA	+++	NA	NA	NA
T66I/Q146P/S147G	NA	+++	NA	NA	NA
T66I/Q95K/Q146P/S147G	NA	+++	NA	NA	NA
T66I/Q95K/E138K/Q146P/S147G	NA	+++	NA	NA	NA
T66I/T97A/E157Q	−	++	−	NA	−
T66I/R263K	−	++	−	NA	NA
T66I/E138K/R263K	+	+++	−	NA	NA
T66K	+	+++	−	−	NA
T66K/L74M	++	+++	+	+	NA
*E92*					
E92G	−	+	−	NA	NA
E92G/S153F	−	+	NA	NA	NA
E92I	−	+	−	−	
E92Q	+	++	−	−	−
V72I/E92Q/E157Q	+	++	NA	NA	NA
E92Q/S147G	−	+	NA	NA	NA
H51Y/E92Q/S147G	NA	+++	NA	NA	NA
H51Y/E92Q/S147G/E157Q	NA	+++	NA	NA	NA
E92Q/E157Q	++	++	−	NA	−
E92Q/R263K	+	+++	−	NA	
E92V	−	+	−	−	NA

Scale: ‘−’ no fold-change (FC), ‘+’ low FC, ‘++’ moderate FC, and ‘+++’ high FC from measured WT 50% inhibitory concentration (EC_50_). NA denotes no value available. Numbers refer to amino acid position in HIV integrase, one letter amino acid code used

References: [14, 16, 28, 29, 31, 32, 36, 40, 43–45, 49–52, 54–69]

**Table 6:** Levels of in vitro resistance for HIV-1 INSTI mutated viruses with raltegravir resistance pathways

Genotype	RAL	EVG	DTG	CTG	BIC
*Y143*					
Y143C	+	−	−	−	−
L68V/Y143C	++	−	−	NA	−
L68V/L74M/Y143C	+++	++	−	NA	−
L74M/T97A/E138A/Y143C	++	NA	−	NA	
Q95K/Y143C	+	−	NA	NA	NA
T97A/Y143C	+++	+	−	NA	−
T97A/Y143C/G163R	++	+	NA	NA	NA
L74M/T97A/Y143G	++	NA	−	NA	NA
Y143H	−	−	−	−	NA
Y143K	+	NA	NA	NA	NA
T97A/E138A/Y143K	++	NA	NA	NA	NA
T97A/Y143S	+	NA	NA	NA	NA
Y143S/V201I	+	NA	NA	NA	NA
Y143R	++	+	−	−	−
V72I/Y143R/T206S	++	NA	NA	NA	NA
T97A/Y143R	+++	++	−	NA	−
L74M/T97A/Y143R	NA	NA	−	NA	NA
L74M/T97A/E138A/Y143R	++		−	NA	NA
L74M/T97A/E138D/Y143R/G163N	+++	++		NA	NA
G140S/Y143R	++	NA	NA	NA	NA

Scale: ‘−’ no fold-change (FC), ‘+’ low FC, ‘++’ moderate FC, and ‘+++’ high FC from measured WT 50% inhibitory concentration (EC_50_). NA denotes no value available. Numbers refer to amino acid position in HIV integrase, one letter amino acid code used

References: [14, 16, 28, 29, 31, 32, 36, 40, 43–45, 49–52, 54–69]

**Table 7:** Levels of in vitro resistance for HIV-1 INSTI mutated viruses with pan-INSTI resistance pathways

Genotype	RAL	EVG	DTG	CTG	BIC
*Q148*					
Q148H	+	+	−	−	NA
G140S/Q148H	+++	+++	+	+	+
E138K/Q148H	++	++	−	−	NA
L74M/T97A/G140S/Q148H	NA	NA	++	NA	NA
L74M/E138A/G140S/Q148H	NA	NA	++	NA	NA
T97A/E138K/G140S/Q148H/N155H	NA	NA	+++	NA	NA
T97A/T112S/G140S/Q148H	+++	NA	+	NA	NA
T97A/T112S/G140S/Q148H/N155H	+++	NA	+++	NA	NA
E92Q/T97A/G140S/Q148H	NA	NA	++	NA	NA
E138K/G140S/Q148H/N155H	NA	NA	++	NA	NA
T97A/G140S/Q148H	+++	+++	++	NA	+
E138A/G140S/Q148H	+++	+++	++	NA	++
E138A/G140S/Y143H/Q148H	+++		++	NA	NA
E138K/G140S/Q148H	+++	+++	++	NA	+
E138K/G140S/Q148H/M154I	+++	+++	+	NA	NA
V75I/E138K/G140S/Q148H/M154I	+++	+++	+	NA	NA
G140S/Y143H/Q148H	NA	NA	+	NA	NA
G140S/Q148H/N155H	+++	NA	NA	NA	NA
T112S/G140S/Q148H/G163K	+++	NA	NA	NA	NA
G140S/Q148H/G163K	+++	+++	+	NA	−
Q148K	+++	+++	−	+	NA
G140S/Q148K	+	+++	−	+	NA
E138K/Q148K	+++	+++	++	+++	+
E138K/G140A/Q148K	+++	+++	+++	NA	+++
Q148N	−	+	−	NA	NA
G140S/Q148N	−	+	−	NA	NA
Q148R	++	+++	−	NA	−
T66I/Q148R	++	+++	+	NA	NA
E92Q/Q148R	+++	+++	+	NA	NA
G140S/Q148R	+++	+++	+	+	+
G140S/Q148R/V201I	+++	+++	+	NA	NA
G140C/Q148R	+++	+++	+	++	−
G140A/Q148R	+++	+++	−	NA	−
G140A/Q148R/G163R	++	NA	NA	NA	NA
E138K/Q148R	+++	+++	+	++	−
E138K/G140S/Q148R	+++	+++	+	NA	NA
E138A/Q148R	++	++	+	++	−
N155H/Q148R	+++	+++	+	NA	+
L74I/G140S/Q148R	+++	+++	NA	NA	NA
L74M/G140A/Q148R	+++	+++	++	NA	+
L74M/G140C/Q148R	+++	+++	++	NA	++
E138A/S147G/Q148R	++	+++	−	+	NA
E138K/G140C/Q148R	+++	+++	++	NA	+
*N155*					
N155H	++	++	−	−	−
T66I/N155H	++	+++	NA	NA	NA
V72I/N155H	++	NA	NA	NA	NA
L74M/N155H	++	++	−	+	−
L74M/V151I/N155H	++	++	NA	NA	NA
L74M/T97A/Y143R/N155H	NA	NA	+	NA	NA
L74M/N155H/R263K	+	+++	+	NA	NA
L74M/T97A/E138A/Y143R/N155H	NA	NA	+	NA	NA
E92Q/N155H	+++	+++	+	+	+
E92Q/V151I/N155H	++	++	NA	NA	NA
E92Q/N155H/G163R	+++	+++	++	NA	+
E92Q/N155H/R263K	++	+++	+	NA	NA
Q95K/N155H	+	++	NA	NA	NA
T97A/N155H	++	++	−	+	−
T97A/V151I/N155H	+++	NA	NA	NA	NA
T97A/V125A/V151I/N155H	+++	NA	NA	NA	NA
T97A/N155H/R263K	+	+++	+	NA	NA
S119R/S147G/V151I/N155H	++	++	−	NA	NA
S119R/T97A/E138K/S147G/V151I/N155H	+++	+++	++	NA	NA
V125A/V151I/N155H	++	NA	NA	NA	NA
E138D/N155H	+	++	NA	NA	NA
Y143H/N155H	++	++	−	+	NA
V151I/N155H	NA	NA	NA	NA	NA
E157Q/N155H	NA	NA	NA	NA	NA
N155H/E157Q/R263K	+	+++	+	NA	NA
N155H/G163K	++	++	−	−	NA
N155H/G163R	++	++	−	−	−
N155H/G163R/R263K	+	+++	+	NA	NA
N155H/D232N	++	++	−	+	NA
N155H/R263K	+	+	+	NA	NA
N155S	+	++	−	−	NA
N155T	+	++	−	−	NA

Scale: ‘−’ no fold-change (FC), ‘+’ low FC, ‘++’ moderate FC, and ‘+++’ high FC from measured WT 50% inhibitory concentration (EC_50_). NA denotes no value available. Numbers refer to amino acid position in HIV integrase, one letter amino acid code used

References: [14, 16, 28, 29, 31, 32, 36, 40, 43–45, 49–52, 54–69]

**Table 8:** Levels of in vitro resistance for HIV-1 INSTI mutated viruses with non-canonical INSTI resistance pathways

Genotype	RAL	EVG	DTG	CTG	BIC
*R263K*					
R263K	−	+	+	−	−
M50I/R263K	−	+	+	NA	+
M50I/S119R/R263K	+	++	+	NA	+
H51Y/R263K	−	NA	+	NA	NA
S119R/R263K	−	+	+	NA	+
E138K/R263K	+	+	+	NA	NA
S153Y/R263K	+	++	+	NA	+
*OTHER*					
M50I	−	−	−	NA	NA
H51Y	−	NA	−	NA	NA
H51Y/S147G	−	+	NA	NA	NA
H51Y/R262K	−	NA	−	NA	NA
V72I	NA	+	NA	NA	NA
V72I/F121Y/T125K	++	++	−	+	NA
V72I/F121Y/T125K/I151V	+	++	−	−	NA
L74M/G118R	++	+	NA	NA	NA
Q95K	+	+	NA	NA	NA
T97A	−	−	−	NA	NA
T97A/F121Y	+++	+++	−	NA	−
L101I	−	NA	−	NA	NA
L101I/S153F	−	−	−	NA	NA
L101I/T124A/S153F	−	−	−	NA	NA
H114Y	−	+	NA	NA	NA
G118R	+	−	+	NA	NA
G118R/E138K	+	−	+	NA	NA
G118S	−	+	−	−	NA
S119R	+	+	−	NA	+
F121Y	++	++	+	−	−
F121Y/T124A	+	+	NA	NA	NA
F121Y/T125K	+	++	−	−	NA
F121Y/G163R	NA	++	NA	NA	NA
T124A	−	−	−	−	NA
T124A/S153Y	−	+	NA	+	NA
T125K	NA	−	NA	NA	NA
A128T	−		−	NA	NA
E138K	−	−	−	NA	NA
G140S	−	+	−	NA	NA
P145S	−	+++	−	−	NA
Q146L	−	++	NA	+	NA
Q146P		+	NA	NA	NA
Q146R	−	+	−	−	NA
S147G	−	+	−	NA	NA
V151I		+	NA	NA	NA
V151L	+	++	+	NA	NA
S153F	−	+	−	NA	NA
S153Y	−	+	+	NA	NA
M154I	−	−	−	NA	NA
E157Q	−	+	−	NA	NA
G193E	−	−	−	NA	NA
S230R	−	NA	−	NA	NA

Scale: ‘−’ no fold-change (FC), ‘+’ low FC, ‘++’ moderate FC, and ‘+++’ high FC from measured WT 50% inhibitory concentration (EC_50_). NA denotes no value available. Numbers refer to amino acid position in HIV integrase, one letter amino acid code used

References: [14, 16, 28, 29, 31, 32, 36, 40, 43–45, 49–52, 54–69]

The Y143 pathway is specific to RAL; it is not selected by any other INSTI (Table 2). This specificity was explained when the crystal structure of prototype foamy virus IN in complex with RAL was solved to show that residue 143 interacts directly with the oxadiazole ring of RAL, forming a π − π stacking interaction that is abrogated when this position is mutated [70, 71]. This is in contrast to changes at positions 148 and 155, which disturb the geometry of the IN active site, thereby disrupting the binding of INSTIs [23]. Interestingly, levels of resistance conferred by changes at position 143 are variable depending on the specific amino acid change involved (Table 6). This phenomenon has been extensively studied and been shown to be true also for the fitness of these variants [46, 53]. The most common substitutions at this position are Y143C and Y143R. They cause low to moderate reductions in RAL susceptibility on their own, but the addition of secondary mutations leads to the levels of resistance being greatly increased (Table 6; see also [53]). Although the Y143R pathway provides the highest levels of RAL resistance, it also has a higher genetic barrier to selection, as the amino acid change requires two nucleotide mutations whereas Y143C/H/S only require one change. Both Y143C and Y143H may transition to Y143R and so these substitutions may reflect an intermediary rather than a final selection [53].

There were numerous reports of emergence of substitutions involving position Q148 in response to RAL pressure both in tissue culture and in patient-derived samples, and this is in line with previous studies (Table 3 and [38, 72, 73]). Early in treatment, substitutions at position Q148 may be seen in isolation, but as they impart a severe fitness cost, they are rapidly compensated for by various secondary resistance mutations as shown in Table 3. In growth competition assays, single Q148X mutants showed a significant reduction in fitness compared with WT viruses in the absence of RAL, whereas viruses containing these same mutations outcompeted WT in the presence of the INSTI. Likewise, as secondary mutations are added to Q148X-containing viruses, they outcompete the single mutants whether RAL is present or not [74]. We can see in Table 7 that as secondary resistance mutations accumulate, the fold-changes in susceptibility to RAL greatly increase as well. This helps to explain why the Q148 pathway dominates in RAL selections.

A N155H pathway is also selected with moderate frequency both in vitro and in vivo in response to RAL (Table 3; see also [16, 75]). This single substitution appears to have a less deleterious effect in terms of the replication of the virus, and as such is mostly only co-reported with one or infrequently two additional secondary substitutions [74, 75]. This observation is also supported by the data collected in Table 7: only one or two additional substitutions are required to provide high levels of RAL resistance. In a study examining the evolution of INSTI resistance substitutions in treated patients over time, it was found that mutations at position 155 were often selected earlier during therapy, and then gradually replaced by changes at position 143 or 148 [76]. This may be due to higher levels of resistance conferred by the 143/148 pathways as compared to N155H.

## Elvitegravir

EVG is a monoketo acid derivative that also demonstrated high specificity for inhibition of HIV IN strand transfer reactions [77]. EVG was developed by Gilead Sciences and approved for use in HIV infected individuals in 2012 [26]. Because EVG is processed by the cytochrome p450 enzyme CYP3A4/5, it needs to be co-formulated with cobicistat to boost plasma concentrations. This permits once daily dosing of EVG [78].

It is evident from both Tables 3 and 7 that RAL and EVG share both the Q148 and N155H major resistance pathways, although from our literature review it appears that the latter is most often reported for RAL. The data compiled in Table 7 clearly show that the levels of resistance conferred by the various mutations of the N155 pathway for EVG are at or above those for RAL and the selection of a greater number of secondary resistance mutations in addition to N155H in patients treated with RAL may be a reflection of this difference. This pathway has also been extensively characterized in terms of EVG resistance by several groups [28, 62, 75]. Although some mutants containing the Y143 pathway displayed moderate levels of resistance against EVG (Table 6), this is most likely due to the secondary resistance mutations present.

The T66 and E92 pathways are predominately selected by EVG, although they do display increased likelihood of selection in vitro as opposed to in vivo (Table 1). As was reported with RAL, there is a dynamism to the temporal selection of EVG resistance mutations that may help to explain these differences. The T66 and E92 pathways are selected earlier under EVG pressure, and are gradually replaced by other pathways, such as Q148X [36, 49, 69]. As can be seen in Tables 5 and 7, substitutions in the T66 and E92 pathways provide moderate levels of resistance to EVG, while the Q148 and N155 pathways provide larger fold-changes in resistance. Many more secondary mutations were also reported for T66X and E92X in vivo than in vitro. While this could be a reporting bias, it could also be reflective of the different requirements for replication under EVG pressure in tissue culture versus in a human host. The S147G pathway is also sometimes selected by EVG in tissue culture and in the clinic but only confers moderate to high levels of INSTI resistance when combined with two or more other resistance substitutions (Tables 4 and 8).

One of the major limitations for EVG has been that it shares a clinically significant resistance pathway at position 148 with RAL [28]. Just as is the case for RAL, significant selection both in vitro and in vivo of the Q148 pathway in response to EVG was observed (Table 3), and this pathway also conferred significant reductions in EVG susceptibility (Table 7). Thus, substitutions at position 148, and the accompanying secondary changes, predominate selections with RAL and EVG.

## Second-generation INSTIs

The relatively low genetic barrier and high degree of cross-resistance among the so called “first-generation” INSTIs RAL and EVG spurred research into the chase for “second-generation” drugs of this class, aimed at retaining efficacy against RAL/EVG resistant variants. There have been four candidate second-generation INSTIs to date. DTG, manufactured by ViiV-Healthcare and GlaxoSmithKline, was approved in 2013 for both treatment-naïve and—experienced patients and is the only second-generation INSTI to be approved to date [79]. MK-2048 showed potent activity against most RAL/EVG resistant variants and did not select for the same substitutions in tissue culture studies but its clinical development was halted due to poor pharmacokinetics. Both CAB and BIC are promising and both are currently in advanced clinical trials [15, 19, 50, 80].

The resistance profile of DTG has been extensively characterized during the past few years (reviewed in [16, 26, 81]). DTG has been shown to have a longer binding half-life to HIV IN than either RAL or EVG, which may help to explain why it maintains activity against most first-generation INSTI resistant variants [61].

It can be seen in Tables 2, 3 and 4 that DTG only sporadically selects for common first-generation INSTI resistance substitutions and that resistance to this compound most often derives from the DTG-specific R263K substitution. Although R263K was seen rarely as a secondary EVG resistance substitution prior to the approval of DTG, it has since been selected by the latter in tissue culture selection studies, and in four INSTI-naïve but treatment-experienced patients undergoing DTG therapy [14, 17, 33, 35, 69, 75, 82]. What is notable about this substitution is that, unlike those discussed for RAL and EVG, the R263K substitution only results in low levels of resistance to DTG. It also has a significant impact on the fitness of the virus, and has yet to be compensated by secondary resistance mutations in tissue culture selections [33, 83]. It has been reported that patients with non-B subtype viruses selected for N155 pathway mutations in response to DTG (Table 3; see also [35]). In vitro, subtype B viruses harbouring this mutation are sensitive to DTG, so these selections may reflect a subtype-specific effect [57].

There are fewer reports on the resistance patterns of CAB, a novel INSTI under development at GlaxoSmithKline. CAB was developed concomitantly with DTG and shares most of its structure; it has the potential to be formulated as a long acting injectable for both pre-exposure prophylaxis and treatment of HIV infection [84]. In the LATTE clinical trial, one patient in the CAB arm did develop a mutation in the Q148 pathway, which suggests that this second-generation INSTI may select for the same mutations as RAL and EVG [15]. In in vitro selection studies, CAB has selected for changes at positions 146 and 153 that could also be selected in the presence of EVG and DTG, respectively (Table 4).

BIC is a more recent second-generation INSTI and as such there is less information available in regard to resistance against this drug. Tissue culture selection studies with BIC performed by Gilead Sciences selected for the R263K substitution in IN, and at an earlier week than occurred with DTG in parallel studies [14]. The fact that BIC selected for R263K may be related to structural similarities between this drug and DTG. So far, the results of a phase II trial of BIC at 48 weeks in HIV infected individuals have been reported and, as yet, there has been no detection of resistance-associated changes in IN [19].

As is shown in Table 7 and been reported previously, the Q148 pathway seems to confer the highest fold-changes in resistance to second-generation INSTIs upon the addition of at least two secondary mutations, and has been selected in a patient failing CAB-based therapy [14, 15, 29, 60]. However, the fold-changes in susceptibility to second-generation INSTIs are almost always below those that have been observed with RAL and EVG. Table 3 notes that the Q148 pathway has yet to be selected for in vitro or in vivo by DTG or BIC, suggesting that decreases in susceptibility with Q148 may only be worrisome in INSTI-experienced patients with Q148 mutations; neither compound appears to select for this pathway on their own when they are used in combination with RTI. Of the other first-generation INSTI resistance pathways, only changes associated with position 155 appear to have any effect on the susceptibility of HIV to DTG, CAB, or BIC, and these changes are relatively low-level (Table 7).

## Experienced patients and selections using resistant viruses

Due to the high degree of cross-resistance between RAL and EVG, neither may be used as salvage therapy for patients failing the other. DTG, however, has been used in select cases in patients failing RAL- or EVG-based therapies. The results of these studies are summarized in Table 9. This strategy was first explored in the VIKING phase II clinical trial, in which 27 highly treatment-experienced patients with drug resistant viruses were switched from their RAL-containing regimens to DTG. At week 24 69% of participants achieved undetectable viral loads, as compared to 88% in treatment-naïve patients in the SPRING-2 trial [17, 43]. Patients with Q148X+ two additional secondary mutations fared the worst in this study, which is in line with the in vitro susceptibility assays reported in Table 7. Interestingly, patients who experienced failure with DTG in this study did so through the accumulation of several RAL/EVG resistance mutations in addition to those present at baseline, and not through the selection of DTG-specific mutations such as R263K. It has been reported that the presence of various first-generation INSTI resistance mutations may be incompatible with R263K [57].

**Table 9:** IN substitutions in viruses isolated from RAL- or EVG-experienced patients subsequently failing therapy with DTG

Previous INSTI	Baseline genotype	Genotype at DTG failure	References
RAL	G140S/*Y143H*/Q148H	*L74I, M/E138A*/G140S/Q148H	[43]
RAL	G140S/Q148H	*L74M, I/T97A*/G140S/Q148H	[43]
RAL	L74M/T97A/E138A/Y143R	L74M/T97A/E138A/Y143R*/N155H*	[43]
RAL	L74M/T97A/Y143R	L74M/T97A/Y143R/*N155H*	[43]
RAL	G140S/Q148H	*T97A/E138K*/G140S/Q148H/*N155H*	[43]
RAL	E138A/G140S/Q148H	*E92Q/T97A*/G140S/Q148H	[43]
RAL	G140S/Q148H	*E138K*/G140S/Q148H/*N155H*	[43]
RAL	E157Q	E157Q	[85]
RAL	G140S/Q148H/N155H	*T97A/T112S*/G140S/Q148H/N155H/*S230N*	[45]
RAL	S119R/S147G/V151I/N155H	*A49P/L68F/T97A*/S119R/*E138K*/S147G/V151I/N155H/*L234V*	[64]
RAL	ND^a^	*G118R*	[34]
EVG	ND^a^	*G118R* ^b^	[34]

Substitutions that differ between baseline and treatment failure are in italics. Numbers refer to amino acid position in HIV integrase, one letter amino acid code used. Raltegravir (RAL), elvitegravir (EVG), dolutegravir (DTG), no change detectable (ND)

^a^Patient had undetectable viral load when switched to DTG monotherapy

^b^Along with multiple polymorphisms in IN (D10E, E11D, S24D, D25E, N27H, V31I, L45Q, I60IM, V72IL, T112L, T124N, T125A, V126L, R127K, V201I, K215N, I220V, N232D, L234I and D286N)

There have been other, infrequent reports of RAL-experienced patients failing DTG salvage therapy. One such patient failed RAL and subsequently DTG with the only known resistance-associated change being E157Q [85]. Although the authors found that the IN derived from this patient was highly resistant to both INSTIs, others showed that the laboratory viral strain NL4.3 containing E157Q was hyper-sensitive to DTG, highlighting the variability that background changes and polymorphisms may introduce into analyses [59]. A different patient failed DTG with a combination of the Q148 and N155 pathways, which is reminiscent of the combinations found in some VIKING patients [45, 86]. A RAL-experienced patient was also reported to have failed DTG with N155 pathway mutations [64].

A G118R mutation was selected by MK-2048 drug pressure, as well as by DTG in certain non-B subtypes of HIV-1 [33, 80, 87]. G118R was also shown to be present in two patients, one previously treated with EVG and the other with RAL, during failure on DTG monotherapy [34]. The selection of G118R in certain settings and not others is most likely due to codon usage at position 118; although rare in certain subtypes of HIV-1, the presence of the GGA (G) codon is favourable to a transition to AGA (R).

Analogous to these sporadic reports of INSTI-experienced patients subsequently failing DTG-based therapies, tissue culture selection studies have used HIV with INSTI resistance mutations to mimic the situation seen in some patient populations. The results of these studies are summarized in Table 10. When viruses with mutations of the Q148 pathway are placed under DTG pressure, they select for additional secondary resistance mutations, similar to what was reported in the patients in the VIKING clinical trial. As expected, viruses containing other primary INSTI resistance substitutions can acquire secondary INSTI resistance mutations under continued selection with either RAL or EVG (Table 10; see also [52]). After 30 weeks of DTG selection, viruses that contained E92Q or N155H at baseline selected for R263K [57]. In another study that lasted only 6 weeks, additional substitutions to E92Q, Y143R or N155H were not detected [52].

**Table 10:** In vitro selections using INSTI resistant viruses

Starting genotype	Drug selection	Genotype (week)	References
E92Q	DTG	E92Q (8), E92Q/*R263K* (30)	[52, 57]
E92Q	RAL	E92Q, *L74M*/E92Q (8)	[52]
E92Q	EVG	E92Q (8)	[52]
E138K	RAL	*T66I/T97A*/E138K/*P142T/G163R* (30)	[88]
E138K	EVG	*G70R*/E138K/*N155H/V249I/R263K* (30)	[88]
*Y143C*	RAL	*Y143R, Y143R/G163R, E92Q/Y143R, G163R/E170A* (8)	[52]
Y143R	DTG	Y143R (8)	[52]
Y143R	RAL	Y143R, *L74M*/Y143R, Y143R/*N155H* (8)	[52]
Q148K	DTG	*E138K*/Q148K (8)	[52]
Q148K	RAL	Q148K, *E138K*/Q148K (8)	[52]
Q148K	EVG	Q148K, *E138K*/Q148K (8)	[52]
*Q148R*	DTG	ND (30)	[57]
Q148R	DTG	*G140S*/Q148R, *G140S*/Q148R/*V201I*, *E138K/G140S*/Q148R (8)	[52]
Q148R	RAL	Q148R, *G140S*/Q148R, *G140S*/Q148R/*V259I*, *L74M/G140S*/Q148R (8)	[52]
Q148R	EVG	Q148R, *E138K*/Q148R (8)	[52]
Q148H	DTG	*G140S*/Q148H, *T97A/G140S*/Q148H, *V75I/E138K/G140S*/Q148H/*M154I* (8)	[52]
Q148H	RAL	*G140S*/Q148H	[52]
Q148H	EVG	*G140S*/Q148H	[52]
G140S	DTG	*V131I/V54I/Q148R*/G140S (30)	[57]
N155H	DTG	N155H (8), N155H/*R263K* (30)	[52, 57]
N155H	RAL	N155H, *G70R*/N155H, N155H/*G163R/D232N*, *S119R*/N155H, *P142T*/N155H/*G163R* (8)	[52]
N155H	EVG	N155H, N155H/*S230K*, N155H/*D232N*, N155H/*E170K*, *G70R/V75I*/N155H (8)	[52]
R263K	RAL	R263K (30)	[88]
R263K	EVG	*M50I/T66I*/R263K (30)	[88]
G118R	DTG	*T66I*/G118R/*E138K* (25)	
G118R	RAL	*T66I*/G118R/*E138K* (30)	[88]
G118R	EVG	*T66I*/G118R/*E157Q* (30)	[88]
H51Y	DTG	H51Y/*R262K* (25)	[89]
H51Y	RAL	H51Y/*G140S/Q148R* (30)	[88]
H51Y	EVG	H51Y/*T66I/S147G/G163R/E170K/D232N* (30)	[88]
G140S/Q148R	DTG	*K14R/H51Y/V54I*/G140S/Q148R (30)	[57]
E92Q/N155H	DTG	E92Q/N155H (30)	[57]
E138K/R263K	RAL	*H51N/T66I/T97A/S119R*/E138K/*Y143H*/R263K (30)	[88]
E138K/R263K	EVG	*M50I/T66I/S119R*/E138K/*S147G*/R263K (30)	[88]
H51Y/G118R	DTG	H51Y/G118R (25)	[89]
H51Y/G118R	RAL	H51Y/*T66I*/G118R (30)	[88]
H51Y/*G118R*	EVG	H51Y/*T66I/S147G/H171R/D232N* (30)	[88]
H51Y/R263K	DTG	H51Y/*E138K*/R263K (25)	[89]
*H51Y*/R263K	RAL	*E138K/Y143R*/R263K (30)	[88]
H51Y/R263K	EVG	*V31I*/H51Y/*E92Q*/R263K (30)	[88]

Substitutions that differ between baseline and final selection are in italics. Numbers refer to amino acid position in HIV integrase, one letter amino acid code used. Raltegravir (RAL), elvitegravir (EVG), dolutegravir (DTG), no change detectable (ND)

Attempts to select further changes to DTG-specific resistance pathways have yielded more nuanced results. R263K-containing viruses are sensitive to RAL and unable to select for additional changes under pressure by this compound unless secondary mutations such as H51Y or E138K are also present. R263K, however, readily selects for EVG resistance (Table 10). This is in line with previous data that identified R263K as a secondary EVG resistance mutation [69]. G118R readily selects both primary and secondary resistance mutations under pressure with all three INSTIs, with or without the initial presence of additional secondary mutations. Lastly, the DTG resistance mutation H51Y facilitates the emergence of other resistance-associated substitutions in selections with all three INSTIs, in agreement with its previously characterized role as a secondary change [67].

## Discussion and conclusions

For the first-generation INSTIs RAL and EVG, the resistance pathways that were selected in vitro were generally predictive of the mutations that would arise in patients failing therapy with these drugs, although the frequencies of primary and/or secondary mutations selected may vary depending on whether in vitro or in vivo results are considered. The picture is not so straight-forward for the newer INSTIs. We have very limited information on resistance against newer INSTIs such as CAB and BIC. Since CAB has selected for the Q148 pathway in vivo, it is possible that the clinical resistance profile of this INSTI will resemble that of the first-generation INSTIs. However, since CAB did not select RAL or EVG resistance pathways in tissue culture, the situation may be more complex. BIC has so far selected for the same substitutions in vitro as DTG and this suggests that this compound might also select for similar pathways as DTG in patients.

Although the most common substitution selected in vitro by DTG, R263K, has also been the most common pathway seen in patients failing DTG, other aspects of tissue culture selection studies with DTG have not been as predictive. One-third of all INSTI-naïve patients reported to have failed DTG to date have done so with the N155 pathway (2/6), even though the N155H substitution alone does not cause large fold-changes in DTG resistance in in vitro assays (Table 7). Part of the explanation may be that the majority of selection studies and in vitro INSTI resistance testing has been performed with subtype B HIV-1. Indeed, the two patients who developed N155H in response to DTG both had non-B viruses. In culture selection studies, non-B viruses predominantly selected the G118R substitution and N155H was not observed [33]. This shows a divergence between the in vivo and in vitro resistance profile of DTG.

In the case of the two INSTI-experienced patients who failed DTG monotherapy with the G118R mutation, the effect of polymorphisms and subtype differences on the selection of INSTI resistance may be important, as the GGA glycine codon can more easily transition to AGA, explaining why arginine is present at a higher frequency in non-B subtypes [34]. Many of the secondary resistance mutations listed in Tables 1, 2, 3, 4 and 9 occur at positions that are considered polymorphic, i.e. dependent on subtype and geographical distribution; this complicates the nature of the selection of these polymorphic changes as a function of INSTI exposure. Any description of transmitted INSTI drug resistance (for a review of the effect of subtype diversity and polymorphisms on HIV-1 INSTI resistance see [37]) must also be thereby complicated. A more accurate portrait of patterns of second-generation INSTI resistance mutations in non-B subtypes, which is increasingly important as access to these medications increases in developing countries, will require that selection studies be conducted more frequently with non-B primary isolates [90].

The Q148 pathway remains the dominant route to INSTI resistance, regardless of the individual compound used. All second-generation INSTIs show lower activity against HIV as secondary mutations of this pathway accumulate, and the results of INSTI-experienced patients on DTG therapy suggest that once present, the sequential selection of further mutations in this pathway will result in greatly diminished susceptibilities to this compound (Table 9; see also [86]). Even though the Q148 substitutions may not be selected by either DTG or BIC in vivo, they remain important for the future of these compounds.

Predictions of which pathways will be important for resistance to second-generation INSTIs, unlike first-generation INSTIs, may not easily follow from in vitro studies. If resistance to these compounds turns out to be due to random genetic changes that are not easily identified, genotyping of patient-derived viruses may not be able to predict treatment outcome when these compounds are employed [37, 86]. Perhaps, the high genetic barrier to resistance of DTG will force HIV to evolve along different mutational pathways in vitro versus in vivo, depending on subtype and baseline polymorphisms.
